# Influence of donor age and donor-recipient age difference on intimal hyperplasia in pediatric patients with young and adult donors vs. adult patients after heart transplantation

**DOI:** 10.1007/s00392-024-02477-4

**Published:** 2024-06-24

**Authors:** Sarah Ulrich, Leonie Arnold, Sebastian Michel, Anja Tengler, Laura Rosenthal, Jörg Hausleiter, Christoph S. Mueller, Brigitte Schnabel, Konstantin Stark, Konstantinos Rizas, Ulrich Grabmaier, Julinda Mehilli, Andre Jakob, Marcus Fischer, Julia Birnbaum, Christian Hagl, Steffen Massberg, Nikolaus Haas, Robert Dalla Pozza, Madeleine Orban

**Affiliations:** 1 Division of Pediatric Cardiology and Intensive Care Medicine, University Hospital, LMU Munich, Munich, Germany; 2 Department of Medicine I, University Hospital, LMU Munich, Marchioninistraße 15, 81377 Munich, Germany; 3 Department of Heart Surgery, Ludwig-Maximilians-University, Klinikum Großhadern, Munich, Germany; 4 German Centre for Cardiovascular Research (DZHK), Partner Site Munich Heart Alliance, Munich, Germany; 5 Present Address: Medizinische Klinik I, Landshut-Achdorf Hospital, Landshut, Germany

**Keywords:** Intimal hyperplasia, Donor age, Optical coherence tomography, Pediatric heart transplantation, Adult heart transplantation

## Abstract

**Aim:**

Optimal selection and allocation of donor hearts is a relevant aspect in transplantation medicine. Donor age and cardiac allograft vasculopathy (CAV) affect post-transplant mortality. To what extent donor age impacts intimal hyperplasia (CAV^IH^) in pediatric and adult patients after heart transplantation (HTx) is understudied.

**Methods:**

In a cohort of 98 HTx patients, 58 pediatric (24.1% with adult donors) and 40 adult patients, we assessed the effect of donor age and donor-recipient age difference (D-R) on the continuous parameter of maximal intima thickness (mIT) in optical coherence tomography. We evaluated their predictive value regarding higher mIT and the prevalence of CAV^IH^, defined as mIT > 0.3 mm, and compared it to established CAV risk factors.

**Results:**

In the overall population, donor age correlated with mIT (*p* < 0.001), while in the pediatric subpopulation, both donor age and D-R correlated with mIT (*p* < 0.001 and *p* = 0.002, respectively). In the overall population, donor age was a main predictor of higher mIT and CAV^IH^ (*p* = 0.001 and *p* = 0.01, respectively) in addition to post-transplant interval, arterial hypertension, and dyslipidemia. In the pediatric patients, dyslipidemia remained a main predictor of both higher mIT and CAV^IH^ (*p* = 0.004 and *p* = 0.040, respectively), while donor age and D-R were not.

**Conclusion:**

While there was an effect of the non-modifiable parameter of donor age regarding maximal intimal thickness, a stronger association was seen between the modifiable risk factor dyslipidemia and higher maximal intimal thickness and CAV^IH^ in both the overall population and the pediatric subpopulation.

## Introduction

Despite improvements in medical therapies for heart failure, there is an increasing number of new heart transplant candidates, including both pediatric and adult patients [1–5]. Therefore, the balance of optimal selection and allocation of donor hearts is a relevant aspect of transplantation medicine.

Donor age is one main parameter for the donor-recipient matching regarding heart transplantation [1]. In adults, the guidelines of the International Society of Heart and Lung Transplantation (ISHLT) recommend the selection of donors aged < 45 years or, if older, without evidence of coronary artery disease, and factors such as estimated survival benefit, availability of organs, and the severity of illness of the recipient need to be included. However, no absolute recommendations of upper age limit exist currently [6]. In children, selected patients can also receive adult hearts. A better understanding of the impact of donor characteristics, particularly age and donor-recipient age difference, on post-transplant outcome of heart transplanted (HTx) patients is therefore highly relevant.

In addition to its association with higher mortality rates after HTx [7], donor age could also have an impact on the development of cardiac allograft vasculopathy (CAV) and has been associated with severe stages of CAV necessitating percutaneous coronary intervention [8]. Importantly, CAV currently remains one of the major causes of late graft loss and mortality after pediatric and adult HTx [9–16]. The relevance of donor-recipient age difference regarding CAV is controversial and could be limited to specific age categories [17–19]. This aspect represents a major gap of knowledge, particularly in pediatric patients with adult donors. Besides, studies assessing the impact of donor age and donor-recipient age difference based their definition of CAV mainly on the angiographic definition of CAV that typically represents later stages of CAV [16, 17, 20–22]. The association of donor age with intracoronary imaging findings of CAV is less understood. Here, the focus was mostly set on the association of donor age with donor-related atherosclerosis [23, 24]. The association of donor age with intimal hyperplasia in CAV (CAV^IH^) in intravascular imaging, particularly in optical coherence tomography (OCT), is understudied [26, 27]. An intravascular ultrasound (IVUS) study performed in pediatric HTx patients showed that donor age (mean age 10.9 ± 13.4 years) was a predictor of median intima-media thickness [28]. To what extent the impact of donor age and donor-recipient age difference could differ from other potentially modifiable CAV risk factors needs to be further determined.

The aims of our study including patients after pediatric and adult HTx were to (1) assess the effect of donor age and donor-recipient age difference on the continuous parameter of maximal coronary intima thickness (IT) as well as on the prevalence CAV^IH^ in OCT, to (2) evaluate whether donor age and donor-recipient age difference are independent risk factors for higher maximal IT and the presence of CAV^IH^, and to (3) evaluate the correlation between donor age and other risk factors of CAV^IH^ as hypertension and dyslipidemia in our overall patient cohort and the pediatric subpopulation, including pediatric and adult donors.

## Methods

### Study population

We analyzed OCT examinations of pediatric and adult HTx patients performed routinely during post-transplant follow-up between December 2013 and October 2019 at the Ludwig-Maximilians University of Munich. Prior to the heart catheterization and OCT examination, all patients or their legal representative were informed about the examination and potential complications and gave written informed consent. The study was approved by the institutional ethical review committee of the Ludwig-Maximilians University of Munich. The investigation conforms with the principles outlined in the *Declaration of Helsinki.*

### Inclusion/exclusion criteria

HTx patients with OCT presenting predominantly with atherosclerotic plaques in the examined vessels or vessels including stents after percutaneous coronary intervention were excluded from the study, because of the difficulty of correct analysis of underlying disease after intervention. Additionally, frames of the OCT examination with inability to analyze > 25% of the frame because of artifacts, presence of large side branch, or insufficient flushing were excluded from analysis [25, 29]. Quadrants with plaques or inability to clearly distinguish intima and/or media were excluded from the measurements.

### OCT acquisition

Use of an OCT catheter (FastView Coronary Imaging Catheter, Terumo Corporation, Tokyo, Japan) for image acquisition according to validated non-occlusive techniques [30] and the Lunawave system (Terumo Corporation, Tokyo, Japan) for intravascular imaging.

### Analysis of OCT sequences

OCT sequences were pseudonymized and digitally stored. Two trained, independent, blinded investigators performed the image assessment using the validated software (QIvus® Medis Program Version 2.5.18. Leiden, The Netherlands). Calibration of the OCT images was obtained by adapting the Z-offset. The segment length without excluded frames was defined as effective segment length.

### Quantification of IT

The routinely used method of circumferential, cross-sectional area measurement of IT is challenging in frames with plaques and the coincident loss of the layered wall architecture [30–32]. Therefore, we used a previously described distance-measuring method per quadrant every 5 mm to optimize the quantification of intima measurements [25, 29, 33]. To distinguish intima and media, the consensus definition characterizing the normal vessel wall by a layered architecture was used [30]. In line with previous studies and international definitions of CAV, we defined a maximal IT > 0.3 mm as CAV^IH^ [33–36].

### Definitions of parameters included into analysis

Arterial hypertension was defined as preexisting diagnosis and antihypertensive therapy. Diabetes mellitus and dyslipidemia were defined as preexisting diagnosis based on the patients’ documents. Post-transplant interval was calculated as the time between the timepoint of the analyzed OCT and HTx (years). The donor-recipient age difference was calculated as donor age–recipient age at HTx in years.

### Statistics

Continuous variables are presented as mean and standard deviation (± SD), and categorical variables are presented as number and percentages. Patients were divided into two groups according to their age at HTx (< 18 years and ≥ 18 years). The two groups were compared using a two-sided *t*-test for continuous variables and a chi-squared test for categorical variables.

The effect of the continuous, time-dependent, non-modifiable risk factors donor age, donor-recipient age difference, age at HTx, post-transplant interval on the continuous parameter maximum IT and the parameter of prevalence of CAV^IH^ were assessed by univariate analysis using either Pearson correlation for two continuous variables or point-biseral correlation and compared using the chi-squared test, as appropriate. Associations were categorized according to previous definitions [37].

A multivariate linear regression model was fitted to find independent predictors of higher maximal IT and a logistic regression model for the presence of CAV^IH^. Covariates were chosen based on expert opinion and results from other studies: post-transplant interval, donor-recipient age difference, donor age, dyslipidemia, hypertension, and donor and recipient BMI were included in the models [11, 16, 38–43]. Because donor and recipient age could be highly correlated in HTx patients, recipient age was included as the donor-recipient age difference, to account for both factors. Recipient sex was included to adjust for gender-dependent differences. Protective medication regarding CAV, such as statin and mammalian target of rapamycin (mTOR) inhibitor therapy, was included into analysis as potential modifiers [44, 45]. Model fit was verified by visually inspecting residual plots, outliers were assessed by cook’s distance, and multicollinearity was tested for with the variance inflation factor. Data were analyzed with R statistics version 4.2.2. (R Core Team, 2022).

## Results

### Baseline characteristics

**Fig. 1:**
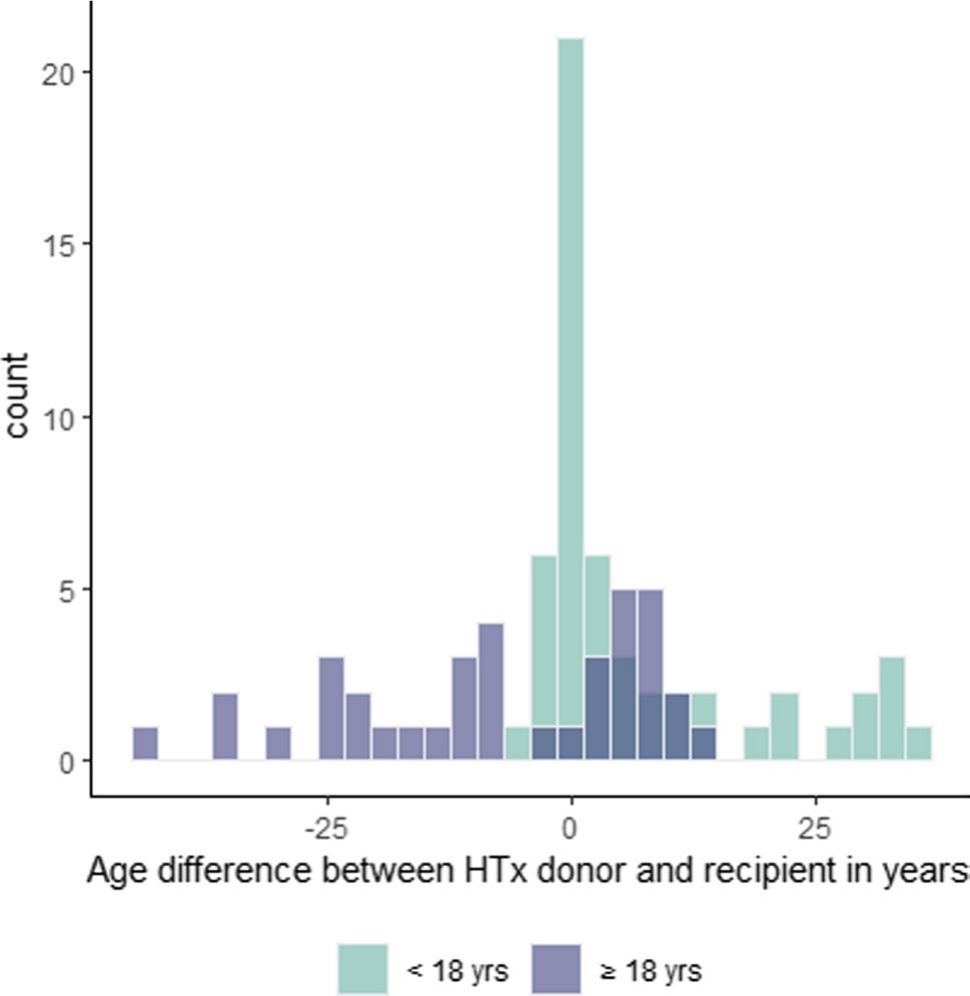
Age difference between HTx recipient and donor by age group. HTx denotes heart transplantation

The baseline characteristics and OCT findings of the overall population and findings according to age category at HTx (pediatric vs. adult patients) are shown in Tables 1 and 2. We included 98 patients into analysis (29.6% female, mean age at HTx 23.7 ± 21.5 years, 59.2% < 18 years at HTx). Donor age was 25.31 ± 19.3 years in the overall population, 40.50 ± 13.0 years in adult patients, and 14.68 ± 15.4 years in pediatric patients. Fourteen pediatric patients received hearts of donors aged > 18 years. Figure 1 represents the donor-recipient age differences in pediatric and adult patients. OCT revealed a prevalence of maximal IT > 0.3 mm in 53.1% of the overall population. Minimal, mean, and maximal IT, as well as the prevalence of IT > 0.3 mm, were higher in patients ≥ 18 years at HTx (*p* = 0.03, Table 2).

**Table 1:** Characteristics of the overall population and according to age category at HTx

Variable	All patients (*n* = 98)	Age category at HTx		
		< 18 years (*n* = 58)	≥ 18 years (*n* = 40)	*p*-value
Recipient characteristics				
Age at OCT (years)	33.04 ± 21.7	17.03 ± 7.2	56.25 ± 12.5	** < 0.001**
Age at HTx (years)	23.65 ± 21.5	7.24 ± 5.9	47.44 ± 11.0	** < 0.001**
Post-transplant interval (years)	9.39 ± 5.7	9.78 ± 5.8	8.82 ± 5.7	0.400
Sex (female)	29 (29.6)	24 (41.4)	5 (12.5)	**0.004**
Recipient BMI, kg/m^2^	21.41 ± 4.6	19.35 ± 4.1	24.48 ± 3.5	** < 0.001**
Recipient CMV reactivation status (yes)	5 (5.1)	2 (3.4)	3 (7.5)	0.3833
Presence of donor-specific antibodies (yes)	9 (9.2)	6 (10.3)	3 (7.5)	0.7308
Donor characteristics				
Donor age (years)	25.31 ± 19.3	14.68 ± 15.4	40.5 ± 13.0	** < 0.001**
Donor-recipient age difference (years)	1.11 ± 15.0	6.95 ± 11.7	-7.27 ± 15.4	** < 0.001**
Sex mismatch (yes)	37 (40.7)	28 (51.9)	9 (24.3)	**0.016**
Donor BMI, kg/m^2^	21.00 ± 6.3	18.10 ± 5.0	25.25 ± 5.5	** < 0.001**
Coronary artery disease (yes)	2 (2.0)	0 (0)	2 (5.0)	0.1641
Cardiac function				
Left ventricular ejection fraction (%)	64.10 ± 4.3	64.60 ± 3.5	63.30 ± 5.1	0.200
Medication				
Prednisolone	5 (5.2)	2 (3.4)	3 (7.7)	0.400
mTor inhibitors	57 (58.2)	44 (75.9)	13 (32.5)	** < 0.001**
Calcineurin inhibitors	85 (86.7)	52 (89.7)	33 (82.5)	0.469
Statin therapy	75 (76.5)	42 (72.4)	33 (82.5)	0.400
Comorbidities				
Arterial hypertension	72 (73.5)	50 (86.2)	22 (55)	**0.001**
Diabetes mellitus	13 (13.3)	6 (10.3)	7 (17.5)	0.500
Dyslipidemia	33 (33.7)	19 (32.8)	14 (35)	1.000
History of acute cellular rejection	18 (18.4)	14 (24.1)	4 (10)	0.100
History of humoral rejection (yes)	2 (2.0)	0 (0)	2 (5)	0.1524

Data are shown as *n* (%) or mean ± SD. *BMI*, body mass index; *HTx*, heart transplantation; *OCT*, optical coherence tomography; *mTor*, mammalian target of rapamycin

**Table 2:** OCT measurements

Variable	All patients (*n* = 98)	Age category at HTx		
		< 18 years (*n* = 58)	≥ 18 years (*n* = 40)	*p*-value
Segment length (mm)	49.50 ± 14.6	51.40 ± 15.2	46.90 ± 13.5	0.100
Minimum IT (mm)	0.01 ± 0.05	0.05 ± 0.04	0.09 ± 0.1	** < 0.001**
Maximum IT (mm)	0.35 ± 0.20	0.31 ± 0.2	0.40 ± 0.2	**0.030**
Mean IT (mm)	0.15 ± 0.09	0.12 ± 0.1	0.19 ± 0.1	** < 0.001**
Maximal IT > 0.3 mm	51 (52.0%)	25 (43.1%)	27 (67.5%)	**0.030**

Data are shown as mean ± SD or *n* (%). *IT*, intima thickness

### Correlation of continuous non-modifiable risk factors with maximal IT

**Fig. 2:**
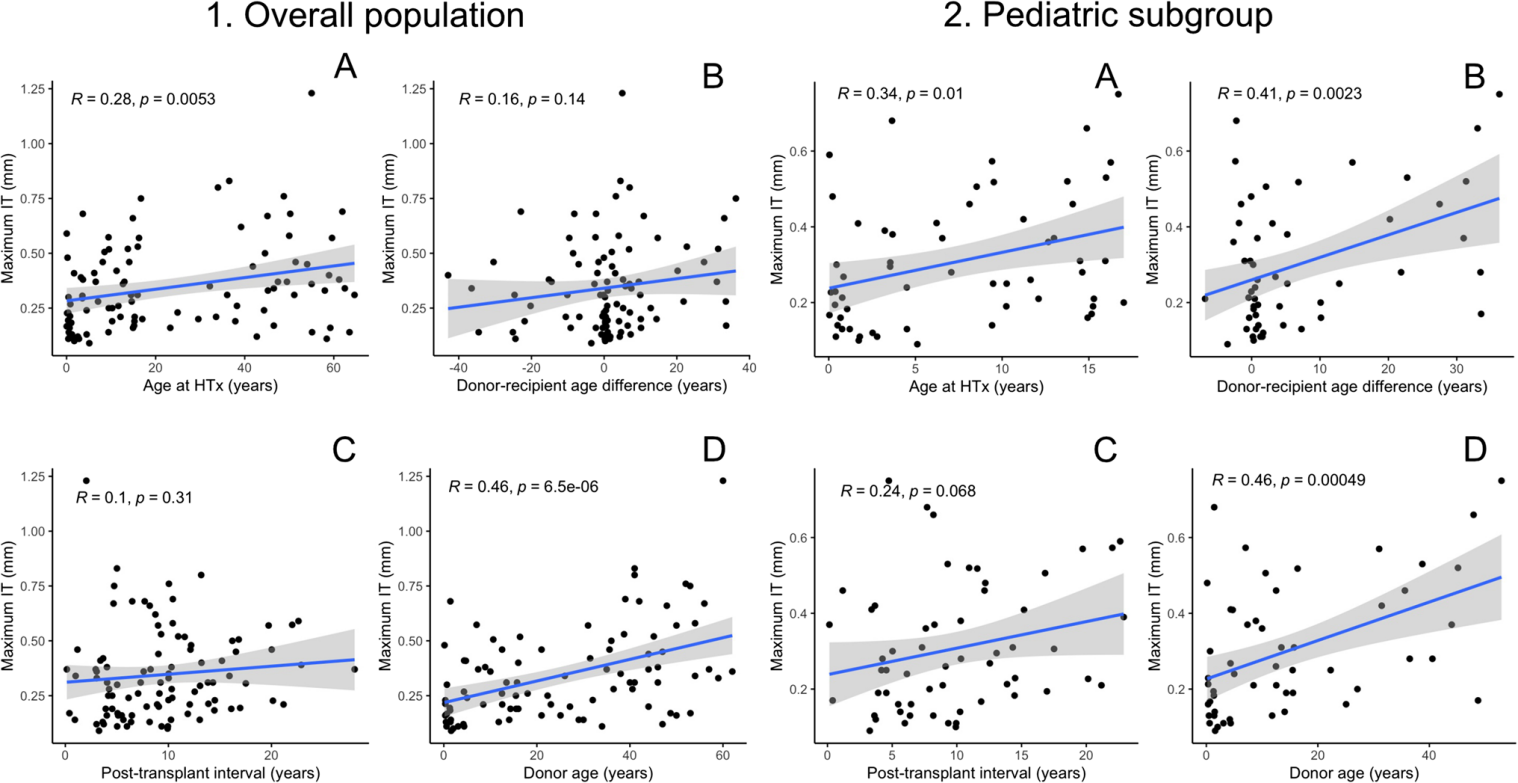
Correlation between maximal intima thickness and time-dependent non-modifiable risk factors for the overall population and for the subgroup of pediatric HTx patients: (**A**) age at HTx, (**B**) donor–recipient age difference, (**C**) post-transplant interval, and (**D**) donor age. HTx denotes heart transplantation

In the overall population, donor age and recipient age at HTx correlated significantly with maximal IT (Fig. 2, *p* < 0.001 and *p* = 0.005, respectively), while post-transplant interval and donor-recipient age difference did not. In the pediatric cohort, donor age (*p* < 0.001), donor-recipient age difference (*p* = 0.002), and recipient age at HTx (*p* = 0.01) correlated significantly with the maximal IT (Fig. 2). There was a trend for a correlation between post-transplant interval and maximal IT (*p* = 0.068).

### Analysis of non-modifiable and modifiable predictors of higher maximal IT

In the multivariable analysis performed in the overall population, donor age (*p* = 0.001), post-transplant interval (*p* = 0.01), arterial hypertension (*p* = 0.03), and dyslipidemia (*p* = 0.02) remained the main predictors of higher maximal IT (Table 3). In the pediatric patients, post-transplant interval (*p* = 0.01) and the presence of dyslipidemia (*p* = 0.004) were the main predictors of higher maximal IT (Table 3).

**Table 3:** Multivariate linear regression model for modifiable and non-modifiable risk factors as predictors of higher maximal intima thickness in the overall population and in the subgroup population of pediatric patients

Predictors	All patients (*n* = 98)			Pediatric patients at HTx (*n* = 58)		
	Beta	CI	*p*-value	Beta	CI	*p*-value
Intercept	− 0.00	− 0.23; 0.23	0.978	0.04	− 0.25; 0.33	0.800
Donor age (years)	0.01	0.01; 0.01	**0.001**	0.01	− 0.01; 0.02	0.070
Donor-recipient age difference (years)	0.00	− 0.01; 0.01	0.200	− 0.00	− 0.01; 0.01	0.800
Donor BMI (kg/m^2^)	− 0.01	− 0.02; 0.01	0.100	− 0.01	− 0.02; 0.00	0.100
Post-transplant interval (years)	0.01	0.01; 0.02	**0.012**	0.01	0.00; 0.02	**0.010**
Recipient sex (male)	0.01	− 0.08; 0.10	0.8	0.01	− 0.07; 0.09	0.900
Dyslipidemia (yes)	0.09	0.01; 0.17	**0.020**	0.13	0.04; 0.21	**0.004**
Arterial hypertension (yes)	0.10	0.01; 0.19	**0.030**	0.06	− 0.06; 0.17	0.300
Recipient BMI (kg/m^2^)	0.01	− 0.01; 0.02	0.500	0.01	− 0.01; 0.02	0.300
mTor inhibitor (yes)	− 0.00	− 0.09; 0.09	1.000	− 0.00	− 0.10; 0.10	1.000
Statin therapy (yes)	0.00	− 0.09; 0.09	0.800	− 0.07	− 0.09; 0.08	0.900
Observations	90			53		
*R*^2^/*R*^2^ adjusted	0.396/0.320			0.547/0.440		

*BMI*, body mass index; *mTor*, mammalian target of rapamycin

### Comparison of non-modifiable risk factors according to the presence of CAV^IH^ (dichotomized)

**Fig. 3:**
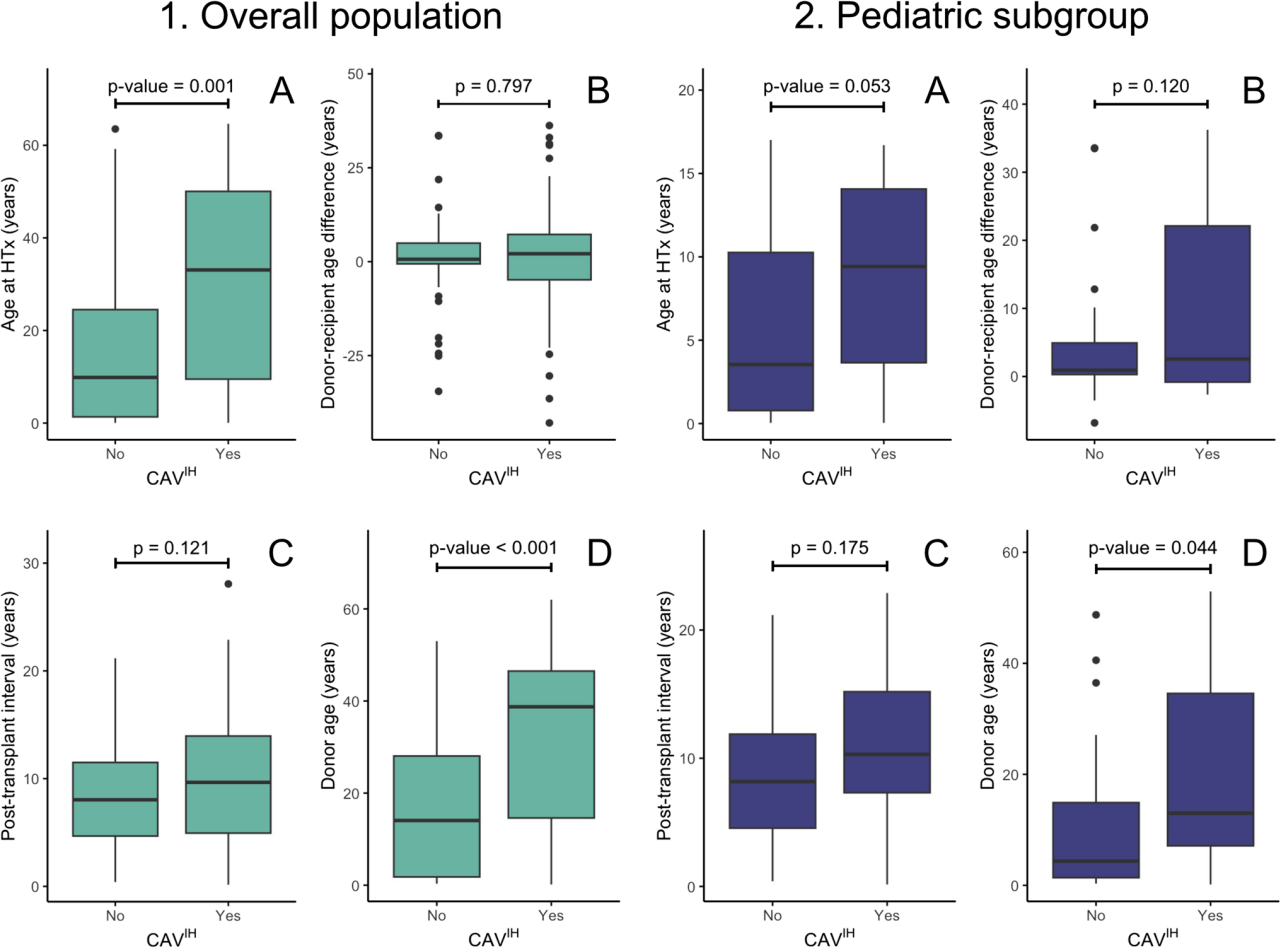
Comparison of non-modifiable risk factors according to the presence of CAV^IH^ in the overall population and for the subgroup of pediatric HTx patients: (**A**) age at HTx, (**B**) donor-recipient age difference, (**C**) post-transplant interval, and (**D**) donor age. HTx denotes heart transplantation, CAV cardiac allograft vasculopathy, CAV^IH^ intimal hyperplasia

In the overall population, patients with CAV^IH^ had a higher age at HTx (*p* = 0.001) and higher donor age (*p* < 0.001), while post-transplant interval and donor-recipient age difference showed no significant difference (Fig. 3). In the pediatric subgroup, patients presenting with CAV^IH^ had a borderline higher age at HTx (*p* = 0.05) and higher donor age (*p* = 0.04), while post-transplant interval and donor-recipient age-difference did not show significant differences (Fig. 3).

### Association of the correlation of modifiable and non-modifiable risk factors with the prevalence of CAV^IH^ (dichotomized)

In multivariable analysis performed in the overall population, the parameters donor age (OR 1.01, 95% CI 1.02; 1.15, *p* = 0.01), post-transplant interval (OR 1.18, 95% CI 1.05; 1.34, *p* = 0.007), arterial hypertension (OR 8.18, 95% CI 1.80; 47.56, *p* = 0.01), and dyslipidemia (OR 9.00, 95% CI 2.50; 40.91, *p* = 0.002) remained significant predictors of the prevalence of CAV^IH^ (Table 4).

**Table 4:** Logistic regression model for modifiable and non-modifiable risk factors as predictors of CAV^IH^ in the overall population and in the subgroup of pediatric patients

Predictors	All patients (*n* = 98)			Pediatric patients at HTx (*n* = 58)		
	OR	CI	*p*-value	OR	CI	*p*-value
Intercept	0.002	0.00; 0.08	**0.002**	0.004	0.00; 2.86	0.100
Donor age (years)	1.08	1.02; 1.15	**0.011**	1.12	0.93; 1.37	0.300
Donor-recipient age difference (years)	1.01	0.97; 1.06	0.600	0.97	0.78; 1.2	0.800
Donor BMI (kg/m^2^)	0.96	0.86; 1.09	0.500	0.86	0.61; 1.09	0.300
Post-transplant interval (years)	1.18	1.05; 1.34	**0.007**	1.10	0.94; 1.31	0.200
Recipient sex (male)	1.78	0.48; 7.02	0.400	1.56	0.33; 7.94	0.600
Dyslipidemia (yes)	9.00	2.50; 40.91	**0.002**	5.81	1.16; 37.80	**0.040**
Arterial hypertension (yes)	8.18	1.80; 47.56	**0.011**	12.56	0.85; 591.93	0.100
Recipient BMI (kg/m^2^)	1.08	0.90; 1.30	0.400	1.19	0.92; 1.60	0.200
mTor inhibitor (yes)	0.59	0.15; 2.39	0.500	0.50	0.07; 3.30	0.500
Statin therapy (yes)	0.96	0.26; 3.57	1.000	0.93	0.19; 4.66	0.900
Observations	90			53		
*R*^2^ Tjur	0.426			0.356		

*BMI*, body mass index; *mTor*, mammalian target of rapamycin.

In the subgroup analysis of pediatric patients, only dyslipidemia (OR 5.81, 95% CI 1.16; 37.80, *p* = 0.04) remained a significant predictor of the prevalence of CAV^IH^ (Table 4) in multivariable analysis.

## Discussion

In our OCT study including pediatric and adult HTx patients,

1. Donor age and donor-recipient age difference had a moderate correlation with the continuous parameter of maximal IT in the pediatric cohort, while only donor age had a moderate correlation with maximal IT in the overall HTx population of our study.
2. In the overall population, donor age, arterial hypertension, and dyslipidemia were independent predictors of higher maximal IT and of the presence of CAV^IH^, while donor-recipient age difference was not.
3. In the pediatric HTx patients, dyslipidemia remained an independent predictor of higher maximal IT and CAV^IH^, while donor age and donor-recipient age difference were not.

Pathology studies have described an impact of age on coronary IT in the general, non-transplanted population, where intimal thickening starts early during childhood [46]. Our findings show that, after HTx, both the ages of recipient and donor influence maximal IT in OCT and are in line with previous studies showing an association of these parameters with angiographic CAV [14, 21]. They extend previous results that suggested that donor age particularly increased the risk of donor-associated atherosclerosis [23, 24].

The effect of donor-recipient age difference regarding the continuous parameter of maximal IT that was only present in the pediatric subgroup might be due to a higher prevalence of older donor hearts in pediatric patients. As opposed, the adult patients of our study had a high percentage of younger hearts, which has been shown to be a protective factor regarding CAV [16]. However, donor-recipient age difference was not an independent risk factor regarding pathological findings of higher maximal IT or the presence of CAV^IH^ in both the pediatric and overall population. These findings are in line with previous results showing no association of donor-recipient age difference with long-term angiographic manifest CAV [17].

Our findings also add important information, as in our study particularly dyslipidemia remained an independent risk factor for higher maximal IT and CAV^IH^. As in our study population, cardiovascular risk factors are typically highly prevalent in HTx patients, probably due to side effects of immunosuppression [47]. Specifically, dyslipidemia is a major side effect of mTOR inhibitors and part of the insulin resistance syndrome that has emerged as a major risk factor of angiographic CAV [48]. Dyslipidemia, and with a milder effect arterial hypertension, have been associated with CAV^IH^ [49–52]. Although there is no evidence for a target LDL concentration, the ISHLT guidelines recommend to aim for LDL levels below 100 mg/dl or less in patients with evidence of CAV [53]. Recommendations regarding statin therapy in pediatric patients are mainly based on adult data showing improved survival after HTx [9]. Our results further strengthen the relevance of dyslipidemia regarding CAV^IH^ in transplanted children.

While our finding suggests that in the overall population, HTx patients with older donor hearts could be at higher risk of intimal thickening, they highlight the relevance of addressing the potentially modifiable CAV risk factor of dyslipidemia [53]. Further studies are needed to define the optimal prevention strategies in the vulnerable population of HTx patients.

## Limitations of the study

In multivariable analysis, the recipient age was represented as the age difference between donor and recipient. This takes the possible influence of a large age gap between donor and recipient into account without having to include the highly correlated variables of donor and recipient age. In histopathological studies, the threshold of IT > 0.3 mm has been defined as pathological. While an association with angiographic lesions is described in HTx patients with CAV, this value has not been prospectively validated in HTx patients [25]. We therefore used the continuous variable of maximal IT and the parameter of higher maximal IT in addition to this cut-off for analysis. The exclusion of patients having undergone percutaneous coronary intervention for CAV could have influenced the results of our study. The pediatric and adult patient group showed a significant difference regarding the number of female patients. The difference found between the overall and pediatric subpopulation regarding the predictive value of arterial hypertension could be potentially explained by the significantly lower incidence of arterial hypertension in the pediatric population, compared to the adult population. Recipient reactivation of cytomegalovirus, history of humoral rejection, the presence of coronary artery disease in the donor, the presence of donor-specific antibodies, and HLA mismatch could have additionally affected findings.

## Conclusion

Maximal intimal thickness, the parameter used to define intimal hyperplasia in CAV, was affected by donor age and donor-recipient age difference in the pediatric patients of our study, while only donor age had a significant effect on maximal intimal thickness in the overall HTx population. The modifiable risk factor dyslipidemia remained a major independent risk factor of higher maximal IT and intimal hyperplasia in both the overall population and the pediatric subpopulation.

## Data Availability

The data underlying this article will be shared on reasonable request to the corresponding author.

## Abbreviations

BMI: Body mass index
CAV: Cardiac allograft vasculopathy
CAV^IH^: Cardiac allograft vasculopathy diagnosed with optical coherence tomography with intima thickness > 0.3 mm
HTx: Heart transplantation
IT: Intima thickness
mIT: Maximal intima thickness
mTOR: Mammalian target of rapamycin
OCT: Optical coherence tomograph
